# “We are the soul, pearl and beauty of Hindu Kush Mountains”: exploring resilience and psychological wellbeing of Kalasha, an ethnic and religious minority group in Pakistan

**DOI:** 10.1080/17482631.2016.1267344

**Published:** 2017-01-09

**Authors:** Fahad Riaz Choudhry, Miriam Sang-Ah Park, Karen Golden, Iram Zehra Bokharey

**Affiliations:** ^a^ Psychology Department, Jeffrey Cheah School of Medicine and Health Sciences, Monash University Malaysia, Bandar Sunway, Malaysia; ^b^ Global Asia in the 21st Century (GA21) Research Platform, Monash University Malaysia, Bandar Sunway, Malaysia; ^c^ Psychiatry Department, Services Institute of Medical Sciences, Lahore, Pakistan

**Keywords:** Marginalization, minority, resilience, interpretative phenomenological analysis, indigenous, Kalash, mental health, mountain people

## Abstract

The Kalasha are a marginalized ethnic and religious minority group in northern Pakistan. The Kalasha minority is known for their divergent polytheistic beliefs, and represents the outliers of the collectively monotheistic Muslim population of Pakistan. This study aimed to explore the psychological resilience beliefs and lived experiences of the Kalasha and to identify cultural protective factors and indigenous beliefs that help them maintain psychological wellbeing and resilience. Seven semi-structured interviews and two focus-group discussions were conducted. The total sample consisted of 6 women and 8 men, aged 20–58 years (*M*_age_ = 36.29, *SD* = 12.58). The Interpretative Phenomenological Analysis qualitative method was chosen. Study findings identified that factors contributing to the wellbeing, happiness and resilience enhancement beliefs of Kalasha included five main themes, all influenced by their unique spirituality: contentment, pride in social identity, tolerance, gender collaboration and gratitude. The study also revealed the Kalasha’s perception of their marginalization related to challenges and threats. The Kalasha emphasized bringing these resilience enhancement beliefs into practice, as a mean to buffer against challenges. In conclusion, this study revealed Kalasha’s wellbeing and resilience enhancement factors, which they believed in and practiced as an element of their indigenous culture and religion.

## Introduction

Southeast Asia and South Asia are among the most culturally diverse parts of the world. However, many of the minority groups and indigenous people in this region are marginalized and receive little government support and legal protection compared to such populations in the West (e.g., Choudhry & Bokharey, 2013; Meijknecht & de Vries, 2010; Miller, 2011). In Asia, ensuring human rights for minorities and indigenous people at the national and regional level is still in its infancy, especially in practice (Ghanea-Hercock, 2004; Hayee, 2012). Moreover, there is a dearth of literature on the ethnic and religious minorities of Asia, and very few studies with a focus on psychological resilience (Choudhry & Bokharey, 2013; Jamadar, 2012; Sharma, 2011).

Psychological resilience provides the ability for groups and communities to survive in the midst of difficult and challenging situations (Hildon, Montgomery, Blane, Wiggins, & Netuveli, 2010; Richardson, 2002). Psychological wellbeing has been defined in terms of physical, affective, cognitive, self, spiritual and social processes (Roothman, Kirsten, & Wissing, 2003). According to Ryff and Keyes (1995) and Shevelenkova and Fesenko (2005), the psychosocial functioning and psychological health of individuals in a community generally reflect the psychological wellbeing of that community. Sagone and de Caroli (2014) found a relationship between resilience and psychological wellbeing by reporting that personal liberty, perception of self-growth and satisfaction was associated with greater resilience.

The current study was conducted in the Bhamburat valley of Kalasha. This region is located in the west of the Chitral district, 2800 metres above the sea and in the midst of the Hindu Kush mountain ranges of northern Pakistan. The Kalasha minority is known for their divergent polytheistic beliefs, and represents the outliers of the collectively monotheistic Muslim population of Pakistan (Williams, 2015). The focal point of inspiration for their cultural identity derives from their spiritual beliefs (e.g., Sheikh, Chaudhry, & Mohyuddin, 2015). The Kalasha historically had little representation in the provincial assembly, and despite amendments to the constitution of Pakistan in 2002, they still have no direct representation in the country’s political system (Malik, 2002). The Kalasha are largely discriminated against in the provision of basic needs, including drinking water, electricity and gas (Zaidi, 2011). The Kalasha culture is endangered for several reasons, including the high rates of Muslim increase in the Kalash Valleys, infant and maternal mortality and the lack of culturally sensitive education for Kalasha children (Malik & Waheed, 2005).

The psychological resilience of this community, however marginalized they may have been, has been noted. For instance, these picturesque valleys have been amongst the worst hit areas by natural disasters, including an earthquake and floods in October 2015. However, when the UNICEF relief team went into help Kalasha when it was struck, they found that the people of Kalasha were extremely resilient emotionally and mentally, despite the challenges they faced (Timme, 2015). While media reports have painted this picture of the community and its people, there has not been a psychological investigation of the exact mechanisms and belief networks that lead them to such resilience. We thus aimed to explore the psychological resilience beliefs and lived experiences of the Kalasha and to identify cultural protective factors and indigenous beliefs that help them maintain psychological wellbeing and resilience. As this minority group faces significant challenges that threaten their survival, it is pertinent to explore their psychological resilience and beliefs.

### Theoretical framework

**Figure 1.**
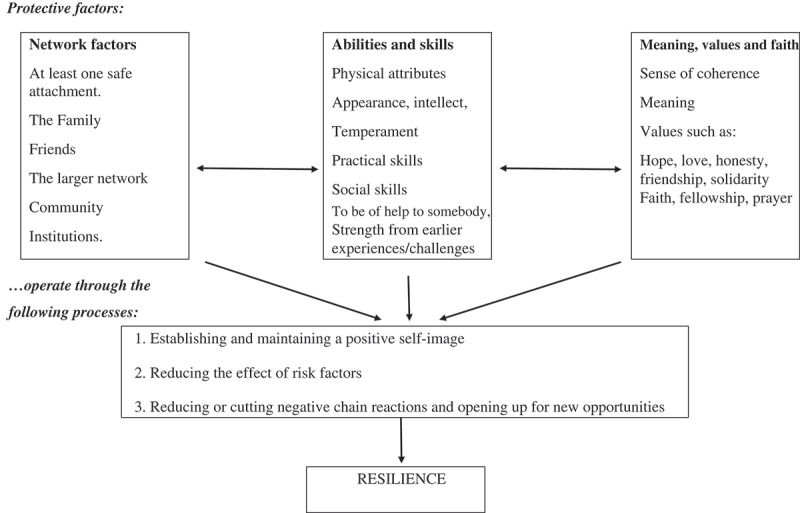
Gunnestad’s (2006) model of development of resilience.

Gunnestad (2006) proposed a model of resilience development by specifically focusing on indigenous populations (Figure 1). This model not only categorized protective factors in three categories (i.e., network factors; abilities and skills; and meaning, values and faith), but also showed that these protective factors combined and promoted resilience development through some psychological processes. By putting forth three cultural factors, Gunnestad (2006) discussed the significance of cultural variables in development of resilience. These three categories work side by side but in their own unique ways, depending on individuals’ situation and infiltrated culture. These three categories also influence one another (Gunnestad, 2006). Resilience arises from building a positive self-image, minimizing the influence of risk factors and breaking a negative circle and bringing new prospects (Gunnestad, 2006). This study aimed to explore the psychological resilience beliefs and lived experiences of the Kalasha and to identify cultural protective factors and indigenous beliefs that help them maintain psychological wellbeing and resilience.

In particular, past studies revealed that the wellbeing of marginalized and minority communities was enhanced when they maintained their cultural values and affinity with their cultural traditions (e.g., Fleming & Ledogar, 2008; Grouzet et al., 2005; Kasser, 2011). For example, holding a strong cultural identity was shown to be associated with a sense of resilience and belonging in minority communities in the USA (e.g., Dockery, 2010; Zimmerman, Ramirez, Washienko, Walter, & Dyer, 1994). Costigan, Koryzma, Hua and Chance (2010) found that stronger ethnic identity was associated with greater achievement and self-esteem, and low depressive symptoms were identified as a strong protective factor against stress, fostering resilience. Furthermore, studies on wellbeing and resilience with marginalized populations have been conducted internationally, and all the findings point to the importance of taking indigenous beliefs into account. For instance, Ritchie, Wabano, Russell, Enosse and Young (2014) focused on resilience and wellbeing of the aboriginal population in Canada, and highlighted the importance of developing culturally appropriate measures of health. Kirmayer et al.’s (2011) study re-conceptualized resilience from an indigenous perspective, and their results revealed that for the Canadian indigenous participants they studied, the concept of resilience was embedded in the concept of their identity, culture, language and traditions. Nystad, Spein and Ingstad’s (2014) study on the community resilience factors of the indigenous community of Norway found interconnectedness among community members and the environment as main factors promoting resilience.

The Kalasha are the last minority tribe having polytheistic beliefs in north Pakistan (Khan, 2008; Trail, 1996). They have maintained their unique traditions, from even before Muslims arrived in the region and, as historians have documented, they have only been marginally touched by the influence of Buddhism and Hinduism (Cacopardo, 2008). The Kalasha are known to be content and cheerful, as well as peaceful, showing gratitude and enjoying their simple pastoralist living (Reddy, 2011). The example demonstrated through the Kalasha may give a deeper understanding of minority communities and their survival, and reveal clues as to how these communities maintain their resilience through times of social change. It would thus be an important task to explore the belief system of this unique group in order to develop an in-depth psychological understanding of their resilient worldviews. We believed that minority status and marginalization do not necessarily lead to lower wellbeing in these communities, contrary to some common beliefs and findings that have reported on the negative impact of rural lives and marginalization on wellbeing (e.g., Alexander, Kinman, Miller, & Patrick, 2003; Cleary, Horsfall, & Escott, 2014; Lynam & Cowley, 2007).

In an ethnographic study, Wynne (2001) described the freedom and liberty of Kalasha women and their openness and freedom in choosing life partners. However, there has not been any study that links such cultural traditions and norms to their wellbeing. We do not have much knowledge as to whether their cultural or ethnic identity affects their wellbeing in a positive or negative way. Also, limited information is available to understand the implications of the intergroup contact they have with the majority groups.Ethier and Deaux (1994), for instance, showed that weaker ethnic identity was related to higher level of perception threat from the environment among Hispanic students, which further lead to a reduction in self-esteem and lower levels of identification with the ethnic group. Therefore, we may argue that if the Kalasha hold a strong ethnic identity and pride with their background, they should be more resilient, regardless of the kinds and strengths of threats they may encounter.

Identity negotiation theory defines identity as the reflective self-images formed, practiced and transferred by people of a certain culture and in a specific communication condition (Ting-Toomey, 2005). Social identity, associated with interdependent self, includes various other aspects of the self-such as one’s social class, disability, sexual orientation, age, cultural or ethnic membership, professional or gender identity (Ting-Toomey, 1999). Cultural identity, a form of social identity, has the importance at the emotional level where an individual associates with the broader culture in which he or she belongs (Ting-Toomey, 2005). There is an association between national identity and state. National identity emerges from nation-building and ideology of a nation. In the same way, national identity arises when an ethnic group focuses on the future and politicizes issues by sharing its homeland (Dahbour, 2002; İnaç & Ünal, 2013; Mandler, 2006).

Jenkins (2008) revealed that identity negotiation occurs in the minds of individuals under various social situations and influences on their lives. For instance, collective or social identities have been shown to manage the anxieties of individuals living in a constantly changing and ambiguous world (Greenberg, Solomon, & Pyszczynski, 1997; Smyth, 2002). Likewise, various researchers have shown a positive relationship between racial or ethnic identity and psychological wellbeing (Fordham & Ogbu, 1986; Lorenzo-Hernandez & Ouellette, 1998; Martinez & Dukes, 1997; Phinney, 1996; Smith, 1991). It was also shown that racial/ethnic identity influenced the self-esteem of only those people who gave utmost importance to race/ethnicity in their identity (Rowley, Sellers, Chavous, & Smith, 1998).

The Kalasha belong to a distinctive cultural, social and religious community placed apart from the majority. Despite the fact that people of Kalasha share the same national identity as other Pakistanis, they may endorse a distinct social and cultural identity arising from the cultural differences; Kalasha practice their own rituals, speak a distinct language and value their own traditions, customs and myths, and this can lead them to endorse and negotiate their identities in a way that may differ significantly from the majority. This is so especially as it seems the Kalasha own and value their cultural or social identity of being a “Kalash group” more than their national identity.

### Aim of the study

The above findings point to the importance of examining the Kalasha’s indigenous beliefs and understanding of resilience. Moreover, given the lack of knowledge, especially in understanding Asian marginalized communities, further investigation is needed. The current study thus aimed to explore the psychological resilience beliefs and lived experiences of the Kalasha, and to identify cultural protective factors and indigenous beliefs that help them maintain psychological wellbeing and resilience. This minority group is reducing in numbers and there is even a possibility that the majority Muslim population will completely overtake them. Despite the marginalization and sociopolitical exclusion, according to Reddy (2011), the Kalasha are often described as the happiest of communities in Pakistan and the most liberated of the Pakistani women (e.g., United Nations International Children’s Emergency Fund, 2015). We aimed to focus on the psychological mechanisms behind their resilience by identifying their perception and interpretation of the challenges and the coping mechanisms they employ to maintain their psychological wellbeing.

## Methods

### Research design

Interpretative phenomenological analysis (IPA) was chosen as the study’s aim was to explore the psychological resilience beliefs and lived experiences of the Kalasha and to identify cultural protective factors and indigenous beliefs that help them maintain psychological wellbeing and resilience. According to Smith (2007), in IPA the researcher tries to understand the participant’s sense making of a phenomenon. In the case of the Kalasha, IPA was viewed as an ideal method for exploring their beliefs about resilience and how they interpret their lived experiences. Smith and Osborn (2003) considered semi-structured interviews as the best and exemplary method for data collection in an IPA study. However, various previous studies (e.g., de Visser & Smith, 2007; Reid, Flowers, & Larkin, 2005) have used focus-group discussions (FGDs) as the basis for IPA. Smith (2004) suggested that the nature of the data is likely to be the key deciding factor as to whether to choose FGDs or individual interviews. Flowers, Duncan and Frankis (2000) used both FGDs and semi-structured interviews in a study and, similarly, this combination was recommended by Dunne and Quayle (2001), who demonstrated that the two methods yielded similar results, hence contributing to the validity of using them in conjunction. The researchers conducted two full-group type FGDs, comprising of 7 participants who were recruited by the researcher on the basis of similar demographic characteristics or attitudes.

### Procedure

Focus group discussions and semi-structured individual interviews were conducted, exploring the beliefs and lived experiences of this marginalized community. After an initial rapport building session, the aims of the study, ethical requirements of getting informed consent and permission of audio recordings were explained to participants. They were also ensured confidentiality. The FGDs were conducted with the help of two moderators, and one translator, who was a local Kalasha who worked as a tour guide, was also present during the FGDs. Two FGDs were conducted with the same participants after a gap of one day. The first focus group lasted 120 minutes plus 40 minutes for initial orientation and engagement with the participants. The second focus group lasted 90 minutes plus 30 minutes for engagement with participants. In second FGD it was revealed that similar responses were appearing, hence, saturation of data was achieved. Therefore no further FGD was conducted. Interview questions/the FGD protocols were developed, with questions based on theoretical concepts of resilience, wellbeing and happiness (see Table 1). During FGDs, all participants were expressive. The FGDs were audio-recorded and a backup audio-recording device was also used. Throughout the process, one moderator was busy making field notes and preparing memos.

**Table 1.** Focus group and semi-structured interview question guide.

Question number	Question
1	Tell us something about Kalasha traditions and culture?
2	How do you describe happiness? What makes you happy?
3a	Tell us about your lifestyle in terms of what are the things that make your lives satisfied?
3b	What are some challenges that you face as a community?
3c	How do you overcome those challenges?
4a	What are adverse events as a community that you have faced in the past or recent past?
4b	How did you overcome/manage those events?
5	What are some barriers to your happiness and wellbeing?
6	How are your interpersonal and intrapersonal relationships?
7a	What are your views about other neighbouring tribes?
7b	What are your perceptions about Muslims?
8	Can you tell me about your life here in the Kalash Valley?

Similarly, following the same pattern of rapport building and ethical requirements, different participants were recruited for semi-structured interview and the interviews were conducted on the subsequent days from the FGDs. Participants were approached through a research gatekeeper (i.e., our guide, a local Kalahsa, who helped spreading the word of our study and also accompanied us to Kalasha’s houses to introduce us). First, three semi-structured interviews were conducted in the houses of the respective participants, as these three females preferred to be interviewed at their own home. However, the remaining four interviews were conducted in a comfortable room in a local hotel, where the environment was conducive and noise was minimal, as this requirement was communicated earlier to the administration of the hotel.

### Participants

The researcher used purposive sampling in order to recruit participants. Purposive sampling is employed when the researcher decides which participants to include in the sample based upon certain criteria (Jupp, 2006). These criteria are based on researcher’s distinct knowledge and capabilities, as well as consent of participants in the study (Jupp, 2006). The participants in this study included six women and eight men, aged 20–58 years (*M*_age_ = 36.29, *SD* = 12.58). For the FGD, nine participants initially agreed to participate, but later two of them changed their mind, leaving seven participants. Similarly, semi-structured interviews were conducted with seven participants. A minimum age criterion was set of at least 18 years. Inclusion criterion was based on the definition of a Kalasha as a person that belongs to the Kalasha tribe, identifies as a Pakistani Kalasha and one who follows its religion and tradition. Exclusion criteria included the non-Kalasha and Muslims living in the same locality and in close proximity to the Kalasha.

### Ethics approval

Ethical requirements were fulfilled as the study was approved by the Research Committee of the Punjab Institute of Mental Health and the District Coordination Officer of Chitral. The local Qazi (a judge practicing religious law) of the Kalasha was also informed about the study. The study also received ethical approval from the Monash University Human Research Ethics Committee. The word was spread through two local hosts/guides regarding study recruitment. The aim and objectives of the study were shared with the participants. They were recruited on a voluntarily basis and were informed that they could withdraw at any time during the discussions. Written informed consent was given by all the participants. No funding was provided for this study. Participant demographics are illustrated in the Results section, along with their individual theme emphasis, following (IPA) data analysis procedures.

### Analytic strategy

First, the IPA strategy required transcription of the recorded data, and then the transcribed data and field notes were read repeatedly. Free textual analysis was conducted (i.e., reading and going through the text a number of times, highlighting phrases to identify a theme or underlying meaning). After this, repeated and similar statements were jotted down together. Those similar statements were then assigned a theme that reflected the psychological mechanism from these similar statements. Once all themes were extracted, the thematic structure was shared with all the authors. Suggestions were given to reorganize themes by authors, and final agreement was reached by consensus of all authors. A double hermeneutic and nomothetic stance was implemented for analysis. Smith and Osborn (2003, p. 51) used the term *double hermeneutic* to emphasize the two interpretations involved in this process: the first is the participant’s meaning-making (interpreting their own experience) and the second is the researcher’s sense-making (interpreting the participant’s account [Reid et al., 2005]). The key theoretical perspectives of IPA are phenomenology, interpretation (hermeneutics) and ideography (Smith, 2004, 2007; Smith, Flowers, & Larkin, 2009).

### Data trustworthiness

Techniques recommended by Elo et al. (2014) and Hill et al. (2005) were used to foster transparency and trustworthiness of data. The same techniques were recommended by Guba and Lincoln (1994) for establishing the credibility of qualitative research. One method was peer debriefing, which involves meetings by the inquirer with a disinterested peer (someone who is willing to ask probing questions but who is not a participant where the study is being conducted) in which the peer can question the methods, emerging conclusions, biases and so on of the inquirer (Hill et al., 2005). This technique helps to independently point out the implications of what the researcher is doing. A peer is typically a person who offers critical questioning regarding the process of research (i.e., data collection) while also reviewing themes (Arber, 2006). An independent peer (not among the study authors) was assigned to this study, going to the field site. Throughout the study, the peer asked questions about data collection and the procedure. He also reviewed the themes and discussed critically how the themes were extracted. His inquiry about every phase of the study kept the researchers alert. He was qualitative researcher himself and lecturer at a local Pakistani university.

Transferability includes rich and thick descriptions and was described by Guba and Lincoln (1994) as a way of achieving external validity. By describing a phenomenon in sufficient detail, one can begin to evaluate the extent to which the conclusions drawn are transferable to other times, settings, situations, and people. It was decided to include even minor details about the methodology and findings in the Methods section. Rich thick descriptions were included in this paper showing verbatim quotes of the participants.

In regards to dependability and conformability, external auditors were assigned for this study. There were also two external auditors (not among the study authors) with whom the data sheets, showing themes and categories, were shared and we requested them to review these and give their feedback. One auditor was an assistant Professor of Psychology in a private university in Pakistan with an expertise in qualitative studies. The second auditor was a Psychology lecturer in another university. Both external auditors suggested few changes in combination and organization of some themes and also renamed a few categories. Those changes were incorporated and the revised draft of themes table was again shared with the auditors and they showed satisfaction with the revision. Finally, the themes were reviewed a couple of times by experienced international researchers also supervising the project (i.e., second and third authors). The supervisors suggested minor revisions and consensus was built on the final version of themes, which was accepted by all authors as well as the independent peer and auditors. Our diverse authorship team, which had members both internal and external to the culture of Pakistan, enhances the trustworthiness of the results by giving their agreement on themes.

## Results

The Kalasha interpreted their lived experiences and revealed their indigenized perspective of resilience that reflects their beliefs and perceptions. The most significant findings of this study fell under five subordinate themes (*contentment, pride in social identity, tolerance, gender collaboration* and *gratitude*), which were under the main superordinate theme of *factors contributing to their psychological* *wellbeing* *and happiness*. The second superordinate theme was *Kalasha’s perceptions of marginalization*. Perceptions of marginalization had two subordinate themes related to *challenges* and *threats*. Furthermore, the *challenges* including *identity challenges* and *lack of support*, while *threats* comprised *religious conversion* and *security needs*. The study further explained Kalasha’s emphasis on practicing the resilience enhancement cognitions and beliefs as protective factors and a way of coping with the challenges and threats they perceived. These are the same five subordinate themes listed above, as identified under the superordinate theme of *factors contributing to* *wellbeing* *and resilience* and influenced through their unique spirituality: *contentment, pride in social identity, tolerance, gender collaboration*, and *gratitude*.

**Figure 2.**
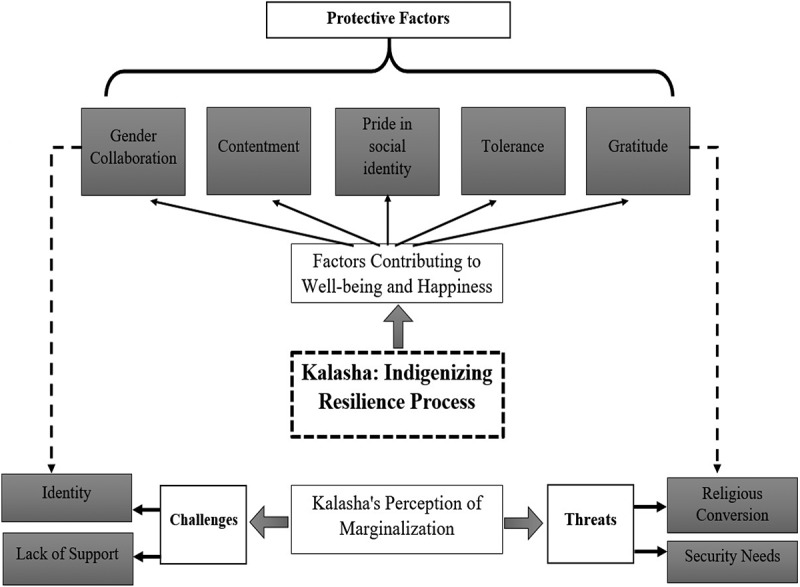
Thematic structure.

A diagram of the thematic structure is illustrated in Figure 2. Table 2 shows more detail of the theme emphasis of the individual participants and also their demographic information.

**Table 2.** Semi-structured interviews and FGD participant demographics and individual theme emphasis.

Pseudonym	Gender	Age	Education level	Marital status	Occupation	Reflections/emphasis
Badshah	Male	42	Matric secondary	Married	Hotel manager	Health, education, conversion of faith, social identity pride and spiritual values
Wali	Male	25	Bachelor’s degree	Married	Private job	Threats of security, education and health, gender collaboration
Palwashey	Female	21	Intermediate	Single	Student	Challenges of education and finances, gender equality, tolerance and peace
Amin	Male	31	No formal schooling	Married	Farmer	Conversion of faith, pride in identity and peace beliefs of love and humanity and challenges
Rashka	Female	37	Primary	Married	Farmer/homemaker	Threats and challenges and overcoming strategies, pride in social identity, gratitude
Jannat	Female	48	No formal schooling	Widowed	Homemaker	Gratitude, spirituality, tolerance and gender collaboration, forgiveness, coping strategies
Gulmir	Male	20	Bachelor’s degree	Single	Student/tourist guide	Kalasha traditions, social identity pride, gratitude, love and peace
Lasib	Male	27	Bachelor’s degree	Single	Development sector	Kalasha exclusion, identity and exlusion, social identity pride and Kalasha documentation, lack of opportunities
Shazia	Female	25	Intermediate	Single	Non-governmental job	Lack of opportunities, gender collaboration
Raqib	Male	37	Matriculation	Married	Shopkeeper	Kalasha traditions, spirituality and tolerance, heaven for visitors
Mirza	Male	54	No formal schooling	Married	Shopkeeper	Peace, identity and exclusion, faith in Kalasha, pride in identity and love for humanity, souls of mountains
Jia	Female	32	No formal schooling	Married	Homemaker	Concepts of purity as a group, belief in multiple gods
GulKhan	Male	51	No formal schooling	Married	Farmer/merchant	Threats from religious extremists, solution in gratitude, spirituality, and faith in Kalasha, identity and exclusion
Marina	Female	58	Primary	Female	Homemaker	Kalasha traditions, tourist heaven, social identity pride, belief in gods

### Factors contributing to psychological wellbeing and happiness

The Kalasha discussed several elements that contribute to their resilience enhancement, wellbeing, happiness and persevering against the challenges.

#### Contentment

Contentment was revealed as a strategy to overcome challenges. The Kalasha believed in adjusting to the changing trends. They realized the importance of education and health; they considered themselves healthy but shared their concern over limited health and higher education opportunities. They showed contentment to these issues but felt the demand for better health and educational facilities. Despite the financial difficulties, the majority of Kalasha agreed that they were not money centric. Badshah expressed his views in these words:

What we will do with money? We have to live here in the heaven of Kalash and not in any other part of world, so we are satisfied, we have enough resources to feed ourselves and our children. The Kalasha are never money oriented, we prefer peace and love that is everything to us.

Jia also showed her views and contentment in following Kalasha traditions when she said:

… we [the tribe] are famous for our faith in purity, we are pure souls like we don’t do negative things, we are satisfied in our life, in our traditions and give no harm to others, we believe that all living peacefully are pure souls.

#### Pride in social identity and tolerance

Beliefs about the Kalasha as a social and religious entity referred to the practices and festivals of thanksgiving to God and cherishing the happy moments. The Kalasha described that their beliefs promote interfaith harmony, peace and love for nature. They believe in nature and that all living and non-living things have a soul and are a reflection of God. They described that holding this belief helps them to promote peace and to stay away from conflicts and aggression. The Kalasha also adopt a national identity, calling themselves Pakistani, which merges with their pride in their religious identity. Palwashey stated:

We are Pakistani, live peacefully with Muslims; our forefathers lived in these Hindukush mountain ranges and were descendants of Alexander. We are proud to be Pakistani.

Raqib, owning Kalasha pride, shared his views as:

We are peace-loving people because we believe in our tradition because we are Kalash and these valleys of Kalash are simply heaven for tourists.

Similarly Badshah added:

We are Pakistani, we own our country, and Kalasha tradition is something which defines Pakistan. We are peace loving and have congenial relations with other tribes and ethnicities.

Wali expressed peace and tolerance as:

We consider Muslims our brothers; we live side by side with them.

The Kalasha strongly believed in giving personal space to each other within their community and to other communities as well, even they don’t hold any reaction grudges against other communities or groups. They have unique spiritual beliefs which taught them tolerance for others and which helped in maintaining a balance within their community. Rashka shared her views in these words:

Every community member has right to live the way he/she wants so if someone does not follow tradition, we do not force them. Similarly, we are not worried much about how to defend our people, so this way we can live with peace of mind instead of reacting.

Badshah stated:

We believe in a live and let live philosophy. … We are free in our choices and we don’t interfere much in each other’s livings, this is why we are confident, strong and we survive against all evils with the blessings of our God Mahadev.

#### Gender collaboration

When the participants were asked about how they overcome the challenges identified, they responded that the collaboration between men and women in every field is a key success element to enhance resilience facing challenges and hardships. They believed in gender equality, which is a very significant finding as the majority of population in the country did not believe in gender equality in the same manner.

As reported by Badshah:

Man and woman are like two wheels of bicycle of life, so they both cooperate mutually to run a family.

Palwashey added:

We do not believe, like others, that women are not allowed to work. They contribute equally, they work with us in fields, shops and we help them in cooking and doing household chores.

Jannat expressed her views in the following words:

We, women, are free and have all the liberty in our lives: we have choice to marry, choice to study or not, and all the major decisions of family and tribe are taken by considering our views and input.

In the same manner Shazia contributed further by showing her agreement she said:

Kalasha women are free in decision making and are not suppressed, Kalash men give respect to women and this gives us courage to face difficulties.

Following such gender equality principles, it can be suggested that their subjective wellbeing also improved likewise.

#### Gratitude

A vital element of resiliency and maintaining peace in Kalasha is their peace loving beliefs as a part of their identity, their love for nature, and their thanksgiving to nature and deities. Badshah reported:

We dance, share wine and enjoy our existence in every season. We arrange a festival for thanksgiving to our gods who blessed us with different seasons and fruits.

Rashka further added to the notion in these words:

Not only showing our thankfulness to God but to pay tribute to our ancestors and to say thanks to each and every person of Kalasha tribe, therefore, all children, young, old, everyone either male or female, participate in these thanksgiving feasts to nature and to people and enjoy singing dancing and music.

Wali reported:

Kalasha is a peaceful community and never shows aggressive behaviour, rather we deal with our conflicts ourselves and if it is beyond our approach then we consult Qazi and we consult our gods by worshipping.

Wali and Palwashey shared their views in the following words:

There are different seasons and different fruits in every season; this is blessing of God.

Amin shed light on their festivals by reporting:

When a child born, it is a celebration and when he dies it is also a celebration as the spirit came in the world and spent good time and finally went back to from where it came from.

Wali reported:

Dancing on death does not mean that we are not sad that our beloved is no more, sadness is something else and happiness dominates it that our beloved’s soul is free.

Similarly, Mirza considered Kalasha as gratitude giving and loving people he said:

We believe in praising and thanking gods for giving us so much, we show our gratitude by arranging functions and by our attitude of gratitude and love for all who are coming here, we are the soul, pearl and beauty of Hindu Kush Mountains. I mean we are center of attraction for people coming here and they enjoy our festivals and culture.

### Perception of marginalization

#### Challenges

##### Identity

One major challenge Kalasha have faced for decades was the exclusion of their identity. They had suffered and faced discriminated largely due to their ethnic and religious identity. For example, they discussed about issues related to their national identity card and passport as Lasib shared:

To give you an example there is no option to select Kalasha as religion when we visit NADRA office for making our national identity cards, there are options for other minorities but Kalasha is not included, same goes with passports.

GulKhan further added, and Mirza seconded him:

This issue was raised some years ago we did lot of efforts of visiting NADRA high officials and bringing this to their notice, also minority minister visited Kalasha who promised to address this issue and just after that they added an option for Kalahsa as a religion in database but this was removed just after few months and the reasons are never revealed to us, despite our many reminders and visits.

Participants have pointed out two opposing viewpoints. A majority of the participants seem to endorse their unique cultural and social identity, which is Kalasha. The responses also reveal the constant peril from ethnic majority groups in the form of religious conversion and oppression as well as the absence of legal rights. However, there are also individuals who seem to acknowledge their national identity and consider the ethnic majority groups, especially Muslims, as their brothers as well. These individuals have signified pride with their national identity and at the same time have given utmost importance to their cultural/social identity.

##### Lack of support

The geographical location in the mountain ranges cut Kalasha from the main urban areas. Hence, they face certain challenges of receiving limited or no development in terms of infrastructure or services within the valleys. They also noted on the financial challenges and non-availability of jobs in this locality, and financial difficulties were expressed as a major challenge. Also, participants knew that health and education were significant elements for the development of any community. However, they also reported and emphasized the *lack of opportunities*. They mentioned that while there were a number of schools for children within their valleys, there was a lack of access to higher education. Also, they were only provided with the very basic health services. As stated by Wali:

Health facilities are very poor here and, for emergencies, we need to travel far away from here.

As Badshah stated:

We also prefer to get earning opportunities, but currently we do not have many options left for us and we prefer not to live outside of our valleys.

When probed about the NGOs there, even though the participants did show some satisfaction with their developmental projects in helping the community more than the government helped, they complained about the lack of job offerings for them to work in the NGOs. A slightly varying view was expressed by Wali:

Foreign NGOs have done many developmental works here on which government never focused; they built schools, library and museum and also sanitation system.

#### Threats

This is the major theme, which appeared in all of the participants’ discussions and is significantly contributing to the existing literature on threat perception.

##### Religious conversion

Kalasha as a community face some threats of religious conversion, including threats of violence from the northern side as well as threats from other groups trying to persuade them to change their religion. The participants reported that Kalasha population is decreasing and near extinction due to these threats. Badhsah reported:

Muslim preachers are working to convert Kalashas and Christians too.

Palwashey further said:

Muslim preachers come, stay in mosques and preach and Christian missionaries come along with NGOs.

Jannat expressed her views in the following words:

Majority is converting to Islam; however, there are Christian missionaries who are giving financial benefits to those who are willing to get converted.

The participants also discussed the impact of these threats as they spoke about those Kalasha who were weak and vulnerable had relocated to some other places, left Kalasha tribes and dispersed, while others converted. GulKhan shared his views by saying:

We have to be grounded and united against these religious extremists’ conversions. We are trying hard to sustain our culture, the solution lies in our values of peace, love and trusting our deities.

##### Security needs

Participants shared that from the last couple of years the security condition has worsened in Kalasha. Previously it was peaceful but the incidents of robbery and snatching were becoming common. Wali said:

Army has started taking care of the security of this area so things are better off now, but such incidents are still reported. My uncle was beaten by dacoits and they put him on gunpoint and took away his animals and money last week.

The increase in such incidents led to action taken by deploying armed forces in the Kalasha surroundings. Since then, there is a marked decrease in such incidents, yet the Kalasha still feel insecure. Badshah expressed:

We are sons of this soil. We are not going to leave this place, our forefathers lived here and Kalasha tradition is in our blood. We are peaceful and love peace and we will not be demotivated or leave this place. Whatsoever is the security condition, we will resist and we will survive here till our last breath.

## Discussion

The aim of this study was to explore the psychological resilience beliefs and lived experiences of the Kalasha and to identify cultural protective factors and indigenous beliefs that help them maintain psychological wellbeing and resilience. Identifying such beliefs will help in developing understanding of resilience in this minority group. Our study yielded outcomes that identified and outlined the main sources of psychological resilience of the Kalasha. The results indicated that the Kalasha, despite the challenges and threats they face, are resilient and hope-filled in the way they perceive their situations as well as the future survival of their community. The Kalasha’s social identity, peace and nature loving attitudes, their freedom of choices in life decisions and their practice of equality for both genders were key findings as factors contributing to their resilience.

The study findings establish that the factors that contribute to Kalasha’s happiness and wellbeing included *contentment, pride in social identity, tolerance, gender collaboration* and *gratitude*. These are the aspects that set the ground for their psychological resilience. We found that their resilience was based on their respect for others, tolerance, unity and pride with their traditional culture. Previous literature demonstrated harmful consequences of identity rejection and prejudice. Some have reported on the long-term effects of rejection, which, if one’s valued identity components are ignored or denied, leads to emotional numbness (Baumeister & DeWall, 2005; Baumeister & Leary, 1995; Richman & Leary, 2009). These findings also relate with the identity negotiation theory (Ting-Toomey, 2015) as the Kalasha take pride in endorsing peculiar social and cultural identities. They try to withstand pressures from other ethnic groups by staying united. However, Kalasha are also proud to be Pakistani, to own their country and to live peacefully with other people. The Kalasha feel bliss because their uniqueness and peculiar beauty is adding to the diversity in Pakistan, which demonstrates that Kalasha are contented of their peculiar social identity as well as national identity. Kalasha’s freedom of choice relates with the empirical findings, suggesting that this autonomy will lead to better wellbeing (Steiner, 1970). Typically, those who display high levels of perceived decision freedom also feel more in control and are less affected by life stressors, and therefore demonstrate more resilience (Gray & Gash, 2014; Lefcourt, 1973; Luthar & Zigler, 1991; Perlmutter & Monty, 1977; Russell, 2016). Such findings reveal that despite of hostile circumstances, the Kalasha were filled with hope and positivity. It seemed that instead of complaining about what they lacked, Kalasha were content with what they have and carried on with their lives with pride. Another important finding was that the Kalasha placed a unique emphasis on giving respect to and empowering women. The Kalasha spoke of women’s rights and their practice of collaborative efforts by men and women, such as how they work together, make decisions with mutual understandings and cooperation, and considered women equally to men in terms of earnings and responsibilities of running a house. Tesch-Römer, Motel-Klingebiel and Tomasik (2007) examined gender differences and subjective wellbeing in different societies and revealed that the overall wellbeing was higher in countries which accept, welcome and encourage gender equality. It seemed that Kalasha are a staunch advocate of gender equality and believe women to be equally significant in every walk of life, which could have contributed to the group’s wellbeing. Therefore, we can conclude that in terms of their resilience enhancement or problem solving, the Kalasha focus on these collaborative efforts and other elements of contentment, pride in their cultural identity, tolerance and gratitude.

It is also important to note that the common and traditional practices of Kalasha played a significant role in their resilience enhancement and wellbeing. At the intrapersonal level, their enigmatic spiritual beliefs influenced their relationships and interactions and expectations within the social settings. Their gestures of paying gratitude to nature and people by celebrating it through music and dancing, as well as practicing “tolerance” and showing satisfaction with their lives in Kalash valleys despite being surrounded by the hard sociopolitical and geographic circumstances, reflects their positive and healthy wellbeing conditions.

These findings are in line with previous studies (e.g., Adger, Huq, Brown, Conway, & Hulme, 2003; DiFulvio, 2011; Nori & Neely, 2009) that revealed that social positions and roles contribute significantly to resilience enhancement, and that tolerance, simplified life styles and contentment also play an important role in resilience growth. Despite the challenges, their identity as a resilient and happy community featured prominently in their discussions. For instance, in discussing the theoretical background for resilience, Richardson (2002) stated resilient qualities, resiliency process and innate resilience as the three primary components. Considering these three theoretical components, the Kalasha’s psychosocial qualities include viewing themselves as peace promoters, gender collaborators and free will practitioners. Their resilience process includes their gratitude and tolerance while facing challenges and threats. Their innate resilience of social pride in their identity and their spirituality and culture are motivational resilience factors. It seemed that the Kalasha are able to maintain their focus on the positive things happening around them and somehow find a meaning to celebrate life, stay united, enjoy every moment of their lives love and respect all human beings equally. Also, the findings of the present study have brought to light various factors that are vital for resilience, similar to what Gunnestad’s (2006) model proposes. This model discussed the significance of indigenous beliefs, meanings and faith in the formation of resilience and similar indigenous beliefs, and the unique spirituality of Kalasha adds fresh insight into this model. The Kalasha are also trying to maintain their own identities and pride with their own unique culture, religious values and geographical location. They are able to ward off the threats they perceive by remaining united and by missing no opportunity of expressing their contentment and gratitude towards God. Despite of absence of job opportunities or higher education, the Kalasha are trying to keep their distinct identity by focusing on what they have and their abilities and skills, and by remaining contended with these. All these factors were shown in Gunnestad’s (2006) model to be significant in resilience building.

### Barriers to psychological wellbeing

Likewise, this study also reflected upon some potential barriers to the psychological wellbeing of Kalasha, which were identified through their perception of marginalization. The Kalasha’s strategies for overcoming these challenges give insight into this indigenous community’s perspective on resilience. While discussing challenges, it was revealed that the major challenges experienced by the Kalasha were to do with “identity,” followed by “lack of government support.” This finding of identity rejection has its significant value in literature, as past literature shows various consequences of rejection. For instance, suffering, negative emotional and behavioral outcomes, negative affect and lowered self-esteem and a state of deprivation lead to detrimental effects on cognition. Also, deleterious effects on health and adjustment in the long run and “hurt feelings” appear as the most predominant negative emotional risks due to rejection (Baumeister & DeWall, 2005; Baumeister & Leary, 1995; Kupersmidt, Burchinal, & Patterson, 1995; Prinstein & Aikins, 2004; Richman & Leary, 2009). The Kalasha’s description of “lack of support” discussing “health challenges” followed by identity challenges are understandable in the context of past findings, which emphasized on links of rejection to poor health outcomes (Baumeister & DeWall, 2005; Baumeister & Leary, 1995; Kupersmidt et al., 1995; Prinstein & Aikins, 2004; Richman & Leary, 2009).

Similarly, the Kalasha people’s perception of threat included “religious conversion” and “security needs.” This threat perception is related to the theoretical framework of threat. Group Threat Theory (Quillian, 1996) states that biased attitudes against an out-group results in higher perceived group threat. According to intergroup threat theory, the perception of harm inflicted by one group over the other group leads to the experience of intergroup threat (Stephan, Oscar, & Morrison, 2009). However, a previous study, by Ethier and Deaux (1994), showed that weaker ethnic identity was related to higher levels of threat perception, which further leads to a reduction in self-esteem and lower levels of identification with the ethnic group. The current study shows that the Kalasha perceive threat, yet they have strong pride in their cultural and ethnic identity. Hence, these contrary findings will be a significant addition to the existing literature on what are considered their coping strategies. The Kalasha also reflected on their “security needs” as they shared that there had been an increase in robbery and dacoit activities in last couple of years with their livestock as the target. However, the most alarming threat for them was religious groups. They divided them into three main groups: (1) “Taliban from the northern side,” specifically referring to the Afghanistan region; (2) “Muslim preachers” and (3) “Christian missionaries working in NGOs.” These findings are in line with a past finding that stated that Kalasha culture is now endangered for several reasons, such as the rates of Muslim influx to the valleys, conversion to Islam, infant and maternal mortality and the lack of culturally sensitive education for the Kalasha children (Malik & Waheed, 2005). The literature suggests that unless immediate measures are taken to preserve the Kalasha culture, the growing majority could potentially overtake the Kalasha minority. This shows that apart from various challenges that the Kalasha face, they are facing grave threats. However, because of their unity, their strong faith and their belief system, the Kalasha are able to ward off the threats related to religious conversion or security needs. The focal point of inspiration for their cultural identity is derived from their unique religious beliefs (Sheikh et al., 2015). According to the participants, the greatest impact of these threats has resulted in the relocation of some Kalasha people and a massive number of Kalasha converting into other faiths, mostly into Islam.

### Managing challenges and strategies to increase wellbeing

The findings indicated that the Kalasha use different techniques or steps toward their resilience building/enhancement, including the practice of gender equality, freedom of choice, holding social identity beliefs, giving gratitude to nature and promoting peace. These strategies have helped them to maintain and build their resilience. The findings of this study are examples for self-help work and are factors that may support optimal human functioning and relate well with the factors considered to act as buffer against mental illness (Seligman, Schulman, DeRubeis, & Hollon, 1999). The findings of this study are consistent with factors that help individuals, communities and societies to flourish in modern times (Seligman & Csikszentmihalyi, 2000). Moreover, their cultural traditions of dancing, singing and celebrating every season by arranging feasts and festivals and sharing sweets, drinks and food with other community members and with tourists are some additional strategies that may help them cherish their identity and maintain their psychological wellbeing.

### Limitations and future research

It should be noted that the study was purely qualitative in its design and has a small sample size. For future studies, a mixed-methods design is suggested in order to improve the generalizability and validity of the findings. Future studies should also target a larger sample, including Kalasha from all three valleys. It is also recommended that separate FGDs are conducted with youth and elders for a better understanding of gender-related views according to age. The health-related beliefs of the Kalasha should also be explored more specifically as this study focused mainly on the psychological outcomes and wellbeing. Some action research into the wellbeing of the Kalasha community is also required. The challenges and resilience enhancement factors identified through this study, like education, health, religious conversion and the psychological impact on their wellbeing, can be explored.

## Implications and conclusion

This study highlighted the key elements and factors that contribute to the resilience building of a community. The identification of cultural protective factors in this group may inform exploration and efforts to foster resilience in other marginalized groups. There are the positive themes highlighted through this study that may be adopted by individuals and groups in terms of resilience building. For example, gratitude, tolerance and gender collaboration may be recommended for resilience enhancement. In clinical settings, the message of this study can be that by taking pride in social identity and by practicing gratitude, one can overcome distress/challenges and can promote wellbeing. The study illustrates the relevance of unique indigenous cultural factors in promoting a community’s resilience.

However, on the basis of the themes extracted from the study, it can be concluded that threats and challenges of Kalasha need to be addressed, with appropriate action taken to provide basic needs to this marginalized community. Studying this population also had its significance as the secondary aim was to explore the psychological resilience beliefs and lived experiences of the Kalasha and to identify cultural protective factors and indigenous beliefs that help them maintain psychological wellbeing and resilience. The recommendations based on the results of this study could be forwarded to the relevant governmental, non-governmental and international institutions for policy making for marginalized groups. Also, the findings of this study are applicable to diverse populations and settings, not limited only to socially excluded groups, but also in the clinical and counselling realm for psychotherapy and counselling, where resilience enhancement is one of the goals set by a patient and the therapist.
